# Does Posterior Capsule Opacification Affect the Results of Diagnostic Technologies to Evaluate the Retina and the Optic Disc?

**DOI:** 10.1155/2015/813242

**Published:** 2015-06-08

**Authors:** Jose Javier Garcia-Medina, Monica del Rio-Vellosillo, Vicente Zanon-Moreno, Enrique Santos-Bueso, Roberto Gallego-Pinazo, Antonio Ferreras, Maria Dolores Pinazo-Duran

**Affiliations:** ^1^Department of Ophthalmology, General University Hospital Reina Sofia, Avenida Intendente Jorge Palacios 1, 30003 Murcia, Spain; ^2^Department of Ophthalmology and Optometry, School of Medicine, University of Murcia, Avenida Intendente Jorge Palacios 1, 30003 Murcia, Spain; ^3^Ophthalmology Research Unit “Santiago Grisolia”, Avenida Gaspar Aguilar, 90, 46017 Valencia, Spain; ^4^Oftared-Retics, Instituto de Salud Carlos III, 28029 Madrid, Spain; ^5^Department of Anesthesia, University Hospital Virgen de la Arrixaca, Ctra. Madrid-Cartagena, s/n, El Palmar, 30120 Murcia, Spain; ^6^Department of Preventive Medicine & Public Health and CIBER Physiopathology of Obesity and Nutrition, School of Medicine, University of Valencia, Avenida Blasco Ibañez 15-17, 46010 Valencia, Spain; ^7^Department of Ophthalmology, San Carlos University Hospital, Calle Profesor Martín Lagos, S/N, 28040 Madrid, Spain; ^8^Department of Ophthalmology, University Hospital La Fe, Bulevar del Sur, 46026 Valencia, Spain; ^9^Miguel Servet University Hospital, Aragon Health Sciences Institute, Paseo Isabel la Catolica, 1-3, 50009 Zaragoza, Spain; ^10^Department of Ophthalmology, University School of Medicine, University of Valencia, Avenida Blasco Ibañez 15-17, 46010 Valencia, Spain

## Abstract

The visual outcome obtained after cataract removal may progressively decline because of posterior capsular opacification (PCO). This condition can be treated by creating an opening in the posterior lens capsule by Nd:YAG laser capsulotomy. PCO optical imperfections cause several light reflection, refraction, and diffraction phenomena, which may interfere with the functional and structural tests performed in different ocular locations for the diagnosis and follow-up of ocular disease, like macular and optic nerve diseases. Some parameters measured by visual field examinations, scanning laser polarimetry, and optical coherence tomography (OCT) have changed after PCO removal. Imaging quality also changes following capsulotomy. Consequently, the results of ancillary tests in pseudophakic eyes for studying ocular diseases like glaucoma or maculopathies should be correlated with other clinical examinations, for example, slit-lamp biomicroscopy or funduscopy. If PCO is clinically significant, a new baseline should be set for future comparisons following capsulotomy when using automated perimetry and scanning laser polarimetry. To perform OCT in the presence of PCO, reliable examinations (considering signal strength) apparently guarantee that measurements are not influenced by PCO.

## 1. Introduction

Phacoemulsification with implantation of intraocular lens in the capsular bag is the most frequent surgical procedure performed in ophthalmology. However, the visual gain obtained after cataract removal may progressively decline due to posterior capsular opacification (PCO) [1].

Despite variations in surgical techniques, intraocular lens material or design, implantation of additional devices, and pharmacological interventions, PCO remains the most frequent long term complication after cataract surgery [1]. Published PCO rates are variable. However, a meta-analysis concluded that approximately 25% of patients operated from extracapsular cataract surgery suffered visually significant PCO within 5 years of the operation [2].

PCO is due to the proliferation of lens residual epithelial cells from the lens equator following cataract extraction, which induces visual alteration by direct interaction with light passing through the visual axis [3]. According to the distribution of proliferation, the resulting opacities may adopt a morphologic pattern or two or even a combination of both: (a) fibrous-type PCO (fibrous epithelial layers) and (b) pearl-type PCO (groups of swollen, optically active, opacified grape-like epithelial growth) [4]. In clinical terms, these changes can diminish visual acuity significantly, alter contrast sensitivity, and cause glare and monocular diplopia [5–7].

Metaplasia of epithelial cells may also induce capsular folds because of mechanical forces. In general terms, these epithelial cells may transform into myofibroblasts, which have contractile properties and allow the posterior capsule to wrinkle [4]. These phenomena may create visual distortions, including a Maddox rod effect, metamorphopsia-like phenomena, or glare [8].

**Figure 1:**
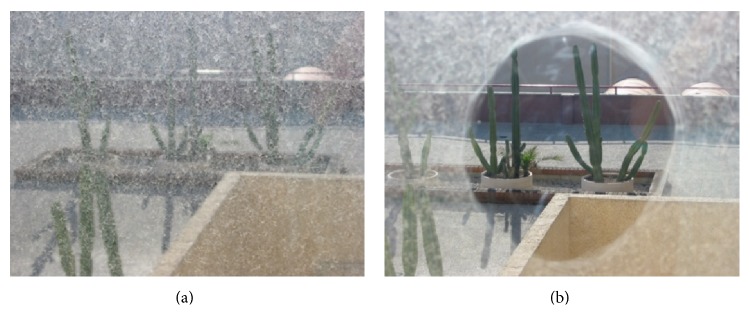
Simulation of vision in a PCO-affected eye before (a) and after Nd:YAG capsulotomy (b).

All of these effects together, these being irregular formations of fibrous proliferation, pearls, and puckers of the posterior capsule generate special properties that affect light reflection, refraction, and diffraction, which may interfere not only with patient vision (Figure 1), but also with functional and structural ocular diagnostic tests [2].

PCO-induced visual affection can be solved by laser Nd:YAG capsulotomy by producing an opening in the posterior capsule and avoiding distortion of light in its passage [1, 4].

An observation made before and after capsulotomy of the outcome of different diagnostic examinations to estimate ocular diseases provides a better understanding of the effects of PCO on such technologies. Recent research suggests that PCO may affect the appropriate ocular disease assessments made by automated perimetry, scanning laser polarimetry, or optical coherence tomography, as seen in Table 1.

## 2. Effect of PCO on Automated Perimetry

A visual field test through white-on-white automated perimetry is a widely used technique and is a useful tool for the diagnosis and follow-up of several ocular disorders, such as glaucoma and neuroophthalmological diseases [9, 10]. Translucent irregularities in the anterior ocular pole, for example, cataracts or PCO, can be a confusing factor that may lead to an inappropriate interpretation of automated perimetry, even when it is not uncommon to encounter patients who are affected or are suspected of being affected, by concurrent entities, for example, PCO and glaucoma or PCO and macular oedema. The ophthalmologist must assess how much visual impairment is due to PCO and how much is related to the other concomitant disorder.

The effect that cataract has on automated perimetry has been well investigated [11–13]. Our research group completed a study in eyes of patients with PCO who underwent a white-on-white automated perimetry test (Humphrey SITA standard programme 24-2) immediately before Nd:YAG capsulotomy and between postsurgery weeks 1 and 8 [14]. The compared pre- and postlaser perimetric indices were mean defect (MD) and pattern standard deviation (PSD).

MD is the average measure of how depressed the patient's visual field is (compared with a control of the same age). Several researchers have reported that MD improved after cataract surgery [11–13]. Similarly according to our results, amelioration of MD occurred after capsulotomy [14].

PSD is a measure of how the different adjacent points on a visual field are. If an area is focally depressed, PSD will rise given the major difference between the points in scotoma and their normal adjacent points. PSD remains unchanged after cataract removal [11–13]. Compared to PCO, however, the PSD in our study improved significantly after capsulotomy. This change could be explained by optical PCO features. PCO translucent opacities apparently induce erratic light-scatter within the eye, which results in a combination of underilluminated retinal areas and in an increased PSD. Yet when these irregularities have been eliminated through capsulotomy, retinal illumination can be more uniform, so PSD lowers [14].

**Figure 2:**
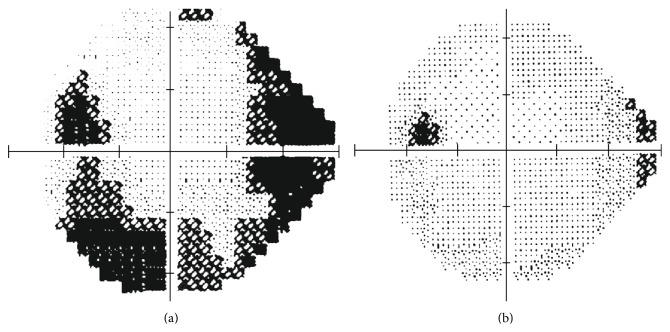
Perimetric defect that mimics inferior arcuate scotoma in a PCO-affected eye (a). The defect partially disappeared after capsulotomy (b).

As the clinical slit-lamp examination aspect can be somewhat guesswork-related and as the automated perimetry analysis corroborates, cataracts depress an automated visual field quite uniformly. So they constitute homogeneous opacities. However, PCOs depress the visual field heterogeneously. Therefore, they have been demonstrated as being polymorphous opacities that may even mimic pathological patterns [14], such as glaucoma arcuate scotoma, which are susceptible to elimination after capsulotomy (Figure 2).

In conclusion, PCO has proven to be a heterogeneous mean opacity. This polymorphism may alter visual field results. For practical purposes, a perimetric defect produced by a PCO can be confused in some cases with a pathologic perimetric defect (false-positive). Consequently, presence of PCO should be taken into account while evaluating any automated perimetry in eyes operated from cataracts.

## 3. Influence of Posterior Capsular Opacification on Scanning Laser Polarimetry

Scanning laser polarimetry (SLP) is a technology for estimating retinal nerve fibre layer (RNFL) thickness* in vivo* at a specific location [15]. It is based on the principle that a polarised laser beam changes its polarisation status when passing through a birefringent tissue. The RNFL is made up of highly ordered parallel axon bundles that contain microtubules, which is the source of its birefringence [15]. As polarised light passes through the RNFL and is reflected back, it undergoes a phase shift. This change in polarisation (retardation), as measured by SLP, correlates with RNFL thickness [16, 17]. Therefore, SLP allows a quantitative assessment of the degree of thinning of the peripapillary RNFL. Such information has been demonstrated as being clinically useful in screening and following up both glaucoma [18–20] and nonglaucomatous optic neuropathies, such as anterior ischaemic optic neuropathy, optic nerve head drusen, and demyelinating optic neuritis [21].

Nevertheless, the RNFL is not the only birefringent structure in the eye. The anterior segment also has birefringent properties, mainly the cornea. Therefore, total retardation of a subject's eye is the sum of both the anterior segment and RNFL birefringence. Accuracy of SLP measurements depends on the ability to isolate RNFL retardation from total ocular retardation [21].

To reduce the effect of anterior segment polarisation, the newest GDx generation incorporates a variable corneal compensator (VCC) that enables compensation of the anterior segment birefringence (ASB) in each individual eye [21].

**Figure 3:**
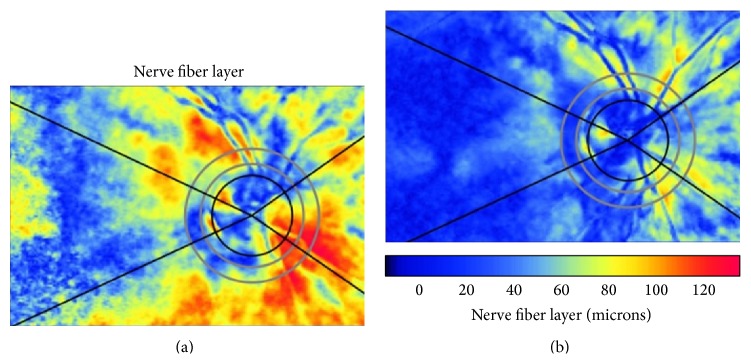
Scanning laser polarimetry examination before (a) and after Nd:YAG capsulotomy (b). Note that the thickness measurements reduce after PCO removal.

Several research works into the effect that PCO and subsequent Nd:YAG capsulotomy have on the SLP results of RNFL retardation measurements have been conducted [22–26]. With this purpose in mind, our research group performed a study into PCO-affected eyes and SLP, selected using GDx VCC, on each patient before and after capsulotomy. We compensated ASB before doing any SLP examination. We compared the SLP parameters before and after PCO removal. We concluded that PCO removal is associated with remarkably significant changes in all the SLP measurements. Briefly, our results suggest that thickness parameters are higher before than after capsulotomy. In other words, SLP examination with GDx VCC may overestimate RNFL retardation measurements in PCO-affected eyes. Therefore, the glaucoma diagnosis in PCO can be underestimated on the basis of the SLP results (false-negatives) (Figure 3) [22, 23]. Furthermore, some SLP measurements (nasal average and nerve fibre indicator) have been significantly associated with best-corrected visual acuity (BCVA) before capsulotomy, which suggests that this technology may be useful for quantifying the degree of PCO [23]. However other authors have not found as many changes in the GDx parameters before and after PCO removal. Vetrugno et al. [24] reported modifications in symmetry, inferior ratio, superior, nasal, and temporal-superior-nasal-inferior-temporal SD whereas Brittain et al. [25] showed significant changes in the typical scan score and temporal-superior-nasal-inferior-temporal average. In addition, Arraes et al. did not show any significant difference between the thickness parameters before and after posterior capsulotomy in patients with moderate degrees of PCO [26]. The variability of the results can be related to the fact that anterior segment birefringence is only assessed before and after laser capsulotomy [23] or only before capsulotomy [25]. The characteristics of the population included in these studies can also be related to this variability noted in the results [23–25].

We also performed a study on a new series of PCO affected eyes that supports our previous conclusions of GDx VCC measurements. In this study we also observed that corneal polarisation axis and corneal polarisation magnitude (the two parameters that determine ASB) changed significantly after PCO removal [27, 28].

Although the results of different studies in the literature are not fully coincident, it is advisable to not only repeat the SLP examination after capsulotomy to serve as a new baseline for the future but also recompensate ASB after Nd:YAG laser application to obtain reliable measurements using GDx VCC.

## 4. Effect of PCO on Optical Coherence Tomography

Optical coherence tomography (OCT) generates high resolution, 2-dimensional cross-sectional images of the internal microstructure of ocular structures. Transverse images of the device are produced using low coherence tomography, an optical measuring technique that is analogous to a B-scan ultrasound, but instead of sound waves, OCT uses a laser-generated beam of light. Two kinds of OCT are available to date: time domain OCT and, more recently, spectral domain OCT. Although both types of OCT use the same basic working principles, the scan rate and axial resolution have improved in spectral domain OCT [29].

OCT explored structures like the peripapillary RNFL and the central retina (including total macular thickness). RNFL thickness, measured by OCT, has been used to study glaucomatous neuropathy, anterior ischaemic optic neuropathy, optic nerve head drusen, demyelinating optic neuritis, traumatic optic tract lesion, Leber hereditary optic neuropathy, and toxic optic neuropathy [21]. Macular assessment by OCT has proved to be a very useful tool for studying the vitreoretinal interface, intraretinal oedema, neuroepithelial detachment, impairments in normal retina architectonics, and its pigment epithelium or choroidal disorders, no matter what its aetiology is [30].

In theory, PCO optical translucent imperfections can alter this beam of light and can, consequently, induce artifactual results in OCT thickness and quality parameters. Several studies have been performed to answer this question.

**Figure 4:**
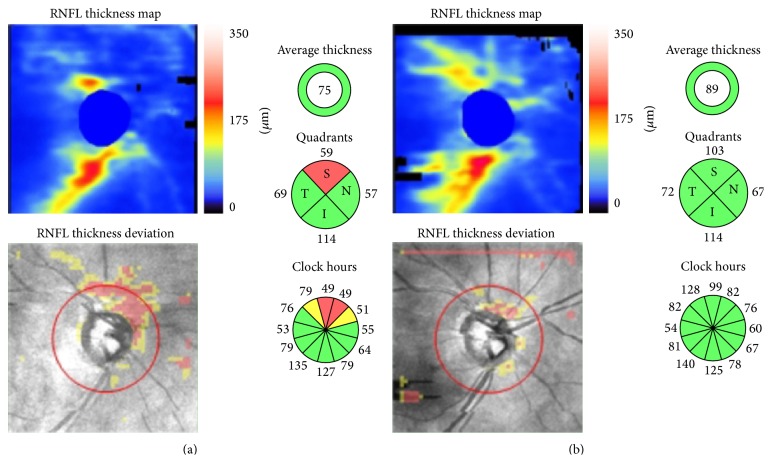
OCT maps of RNFL thickness before (a) and after (b) capsulotomy. Note that thickness measurements increase in the top half of the map after PCO removal.

In relation to peripapillary RNFL measurements, Kara et al. [31] recently investigated the effect that PCO has on the results of RNFL thickness measured by time domain OCT (Stratus, Zeiss). These authors divided eyes into groups according to each signal strength (SS) value obtained, including unreliable (SS < 5) and reliable examinations (SS > 5). They also compared the thickness in each group independently and observed that the lower the SS value, the greater the precapsulotomy RNFL average underestimation and the more significant the results. They concluded that RNFL thickness is affected by PCO. Our group previously carried out a similar study with time domain OCT (Stratus, Zeiss) [32, 33]. We also obtained a significant increase in SS but found no changes in RNFL thicknesses after capsulotomy in reliable scans. When considering all the scans (reliable: SS ≥ 6 and unreliable examinations: SS < 6), we concluded that PCO induces an underestimation of RNFL thickness parameters (Figure 4) as measured by spectral domain OCT (Cirrus, Zeiss). However when analyzing only reliable examinations, no changes between pre- and postlaser measurements were observed [34]. This finding suggests that the prelaser SS value may orientate the degree of reliability of the results, as previously described by Kara et al. [31].

**Figure 5:**
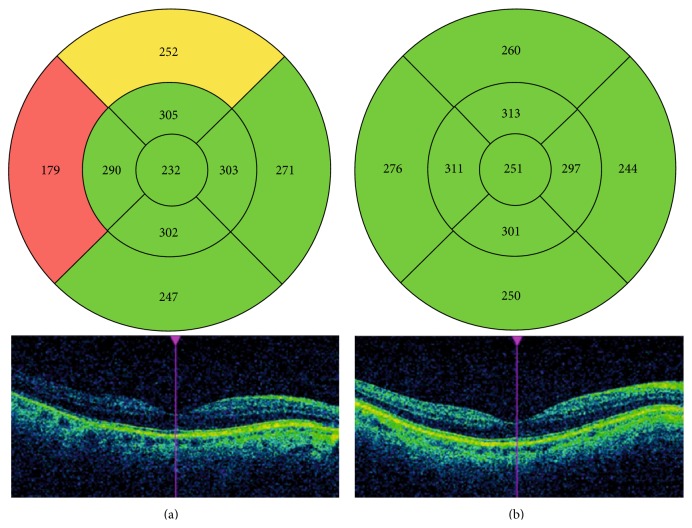
OCT maps of total macular thickness before (a) and after (b) the capsulotomy. Note that most of the thickness measurements increase and image quality improves after PCO removal.

As far as the central retina is concerned, macular thickness parameters have also been compared before and after Nd:YAG capsulotomy in several studies in order to directly assess OCT performance or to indirectly check the safety of the procedures for evaluating macular cystoid oedema as a complication of PCO removal. Most research has shown in both the short and long term that macular thickness parameters, as measured by OCT, have not been seen to change after Nd:YAG laser capsulotomy [35–39]. In one study by our group performed with time domain OCT (Stratus, Zeiss), we concluded that OCT image quality is influenced by PCO. Nd:YAG capsulotomy results in a measurable improvement in quality and improves the number of valuable examinations. However, valuable OCT scans in patients with PCO did not show changes in macular thickness measurements, not even in the presence of severe PCO [40]. In a more recent study using spectral domain OCT (Cirrus, Zeiss), we concluded that all the parameters in the comparisons thickness were higher after capsulotomy than they were before (Figure 5). Yet when we considered only patients with a signal strength of ≥6 (reliable scans), no significant differences were observed in the measurements taken before and after PCO removal [41].

## 5. Additional Comments

Some other concerns should be taken into account when considering the influence of PCO on the above-mentioned tests. Firstly, PCO may have different patterns, that is, central, paracentral, and diffuse ones, which may differently affect the quality and the results of functional and structural tests. The example of the visual field may reflect a paracentral PCO (Figure 2). However to the best of our knowledge, this fact has not been considered in studies to date. Secondly, some data indicate the relation between degree of PCO and test abnormality. Degree of PCO could have also been estimated directly by BCVA. In one study on automated perimetry, the correlation results revealed that BCVA, MD, and PSD were significantly associated both before and after capsulotomy [14]. Another study showed some SLP measurements associated significantly with BCVA before capsulotomy, which indicates that this technology may be useful for quantifying degree of PCO [23]. In relation to OCT, the correlation found between BCVA and SS before capsulotomy suggests that SS could be considered an objective indicator of degree of PCO [40]. Kara et al. [31] also found a significant correlation between, on the one hand, preoperative BCVA and SS and, on the other hand, between preoperative BCVA and degree of PCO. Finally, differences between instruments may be due to, at least in part, differences in inclusion criteria between studies and characteristics of included eyes.

## 6. Conclusion

Optical translucent imperfections of PCO induce special properties relating to reflection, refraction, and diffraction that may alter the ancillary tests used in the diagnosis and follow-up of different optic nerve diseases.

In fact the results of automated perimetry and SLP have been shown to change after capsulotomy. In addition, OCT quality imaging of RNFL thickness is influenced by PCO. However, no change has been observed after PCO removal in the retinal nerve fibre layer parameters of pseudophakic eyes by reliable examinations before capsulotomy, as measured by OCT.

Thus, features of ancillary tests in pseudophakic eyes for studying optic nerve diseases should be well-interpreted and should correlate with other clinical examinations, such as slit-lamp biomicroscopy. If a clinically significant PCO is detected, new measurements should be considered after PCO removal to serve as a baseline for future comparisons, especially when using automated perimetry and SLP. As for OCT in the presence of PCO, reliable examinations (considering signal strength) apparently guarantee that the measurements taken before and after capsulotomy are similar.

## Figures and Tables

**Table 1:** Studies considering the influence of PCO on test results.

Author (year) [reference number]	Test	*n*	Precapsulotomy BCVA (mean ± SD)	Postcapsulotomy BCVA (mean ± SD)	Results after capsulotomy
García-Medina et al. (2006) [14].	AP	26	0.35 ± 0.11 (decimal scale)	0.84 ± 0.14 (decimal scale)	MD and PSD improved.

García-Medina et al. (2006) [23]	SLP	28	0.41 ± 0.12 (decimal scale)	0.85 ± 0.13 (decimal scale)	NFI and TSS increased. Significant decreases of all absolute parameters.

Vetrugno et al. (2007) [24]	SLP	158	0.3 ± 0.6 (LogMar)	0.05 ± 0.2 (LogMar)	Inferior ratio and TSNIT SD decreased. Superior/nasal increased.

Brittain et al. (2007) [25]	SLP	20	0.32 ± un (LogMar)	0.14 ± un (LogMar)	TSS and TSNIT SD increased. TSNIT score decreased.

Arraes et al. (2008) [26]	SLP	37	0.2 ± un (decimal scale)	0.8 ± un (decimal scale)	No significant difference between parameters.

Hougaard et al. (2001) [35]	TD-OCT	13	0.29 ± un (decimal scale)	0.39 ± un (decimal scale)	Signal-to-noise ratio increased but no changes in macular thickness.

Garcia-Medina et al. (2007) [32]	TD-OCT	32	0.25 ± 0.17 (decimal scale)	0.77 ± 0.22 (decimal scale)	SS increased but no changes in pRNFL thicknesses (in reliable exams).

González-Ocampo-Dorta et al. (2008) [40]	TD-OCT	32	0.25 ± 0.17 (decimal scale)	0.77 ± 0.22 (decimal scale)	SS increased but no changes in macular thicknesses (in reliable exams).

Altiparmak et al. (2010) [36]	TD-OCT	54	0.47 ± 0.3 (decimal scale)	0.91 ± 0.14 (decimal scale)	No change of the foveal thickness.

Giocanti-Aurégan et al. (2011) [37]	TD-OCT	30	0.6 ± 0.3 (LogMar)	0.1 ± 0.3 (LogMar)	No change of the foveal thickness.

Wróblewska-Czajka et al. (2012) [38]	TD-OCT	55	NA	NA	No change of the central macular thickness.

Kara et al. (2012) [31]	TD-OCT	98	0.49 ± 0.28 (LogMar)	0.09 ± 0.11 (LogMar)	SS and pRNFL thicknesses increased.

Garcia-Medina et al. (2013) [34]	SD-OCT	37	0.27 ± 0.19 (decimal scale)	0.83 ± 0.18 (decimal scale)	All pRNFL thickness parameters increased. No changes when considering reliable examinations.

Garcia-Medina et al. (2013) [41]	SD-OCT	35	0.23 ± 0.28 (decimal scale)	0.81 ± 0.16 (decimal scale)	All macular thickness parameters increased. No changes when considering reliable examinations.

Ruiz-Casas et al. (2013) [39]	SD-OCT	31	0.4 ± NA	0.8 ± NA	No change of the foveal thickness.

BCVA: best-corrected visual acuity, SD: standard deviation, AP: automated perimetry, MD: mean deviation, PSD: pattern standard deviation, SLP: scanning laser polarimetry, NFI: nerve fiber indicator, TSS: typical scan score, TSNIT: temporal-superior-nasal-inferior-temporal, NA: not available, TD-OCT: time domain optical coherence tomography, SD-OCT: spectral domain optical coherence tomography, SS: signal strength, and pRNFL = peripapillary retinal nerve fiber layer.
